# Supplementary figures and images for: Density of CD3+ and CD8+ cells in gingivo-buccal oral squamous cell carcinoma is associated with lymph node metastases and survival
Source: PLoS One. 2020 Nov 19;15(11):e0242058. doi: 10.1371/journal.pone.0242058 (PMC7676650; doi:10.1371/journal.pone.0242058)

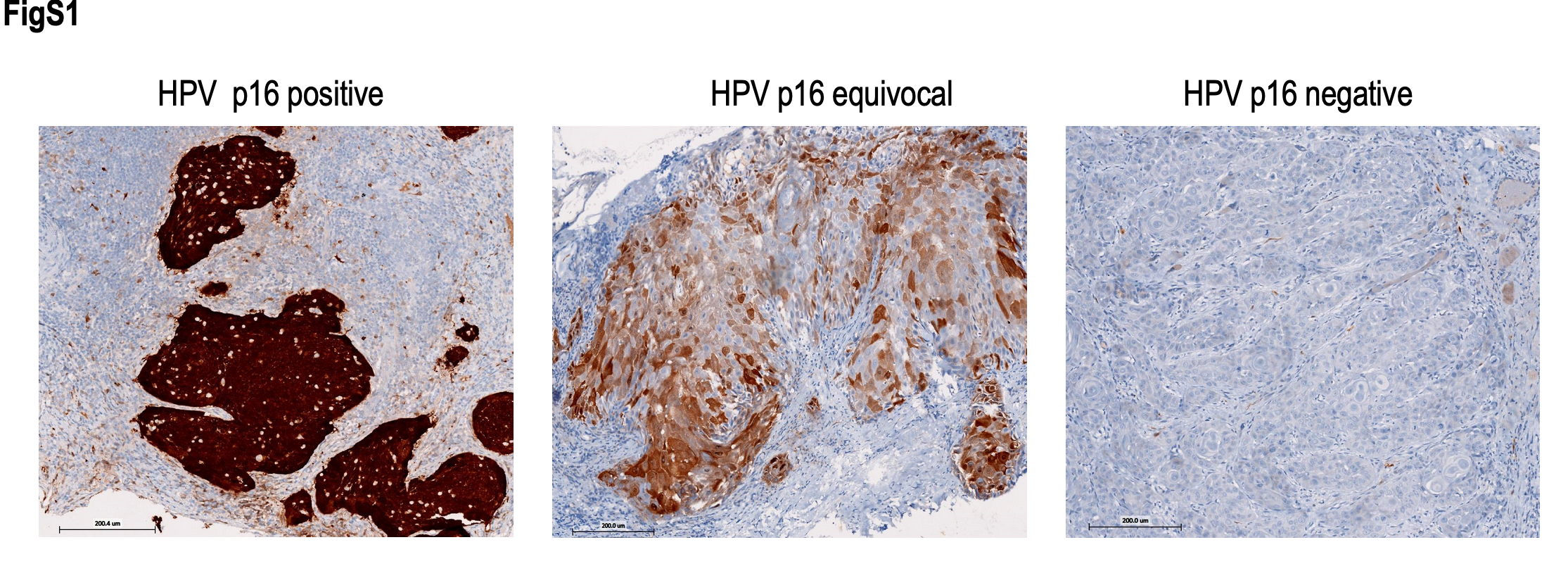

Supplement: S1 Fig — Representative sections show positive, equivocal and negative staining for p16. (TIF) [file pone.0242058.s001.tif]

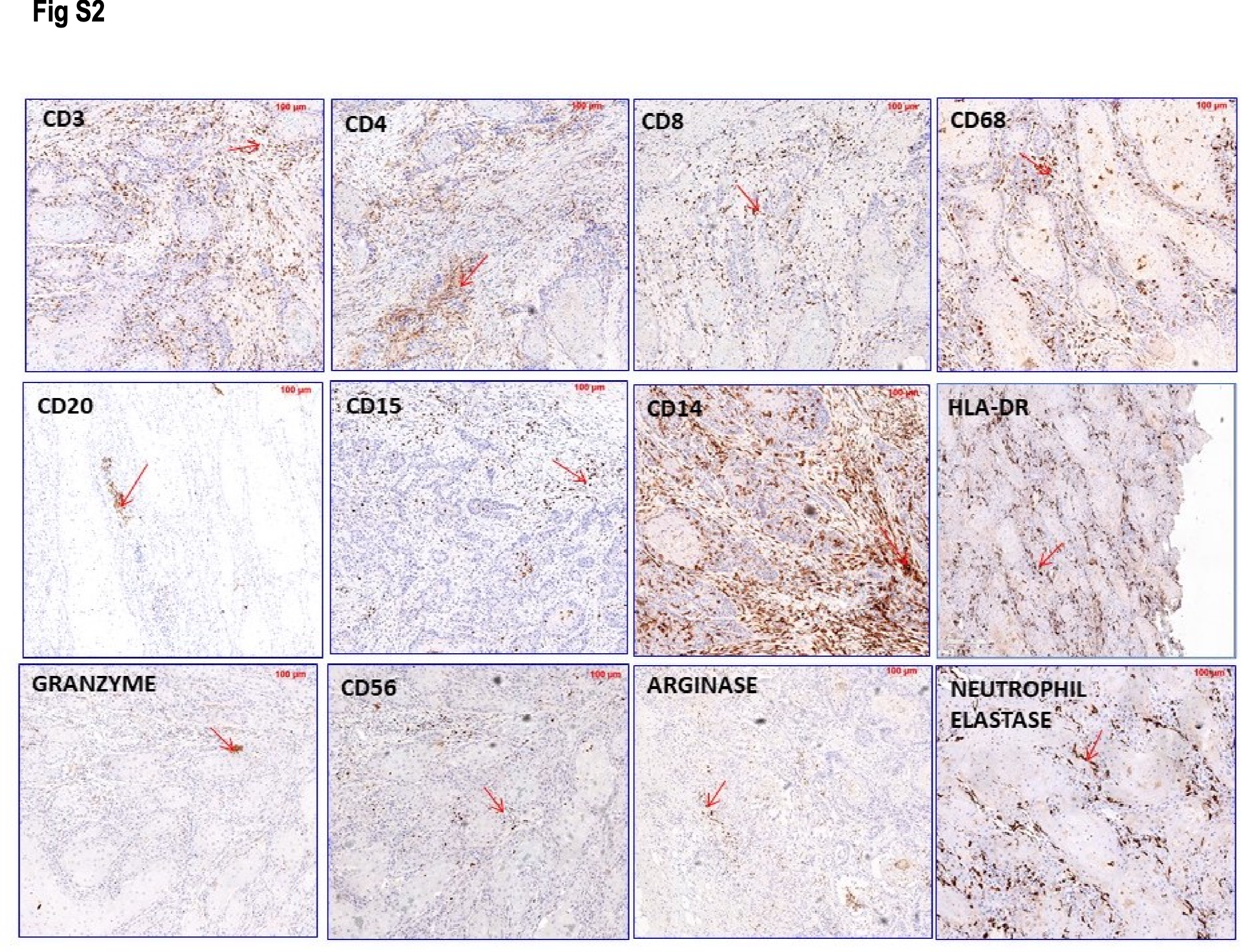

Supplement: S2 Fig — Red arrows indicate the expression of the markers. (TIF) [file pone.0242058.s002.tif]

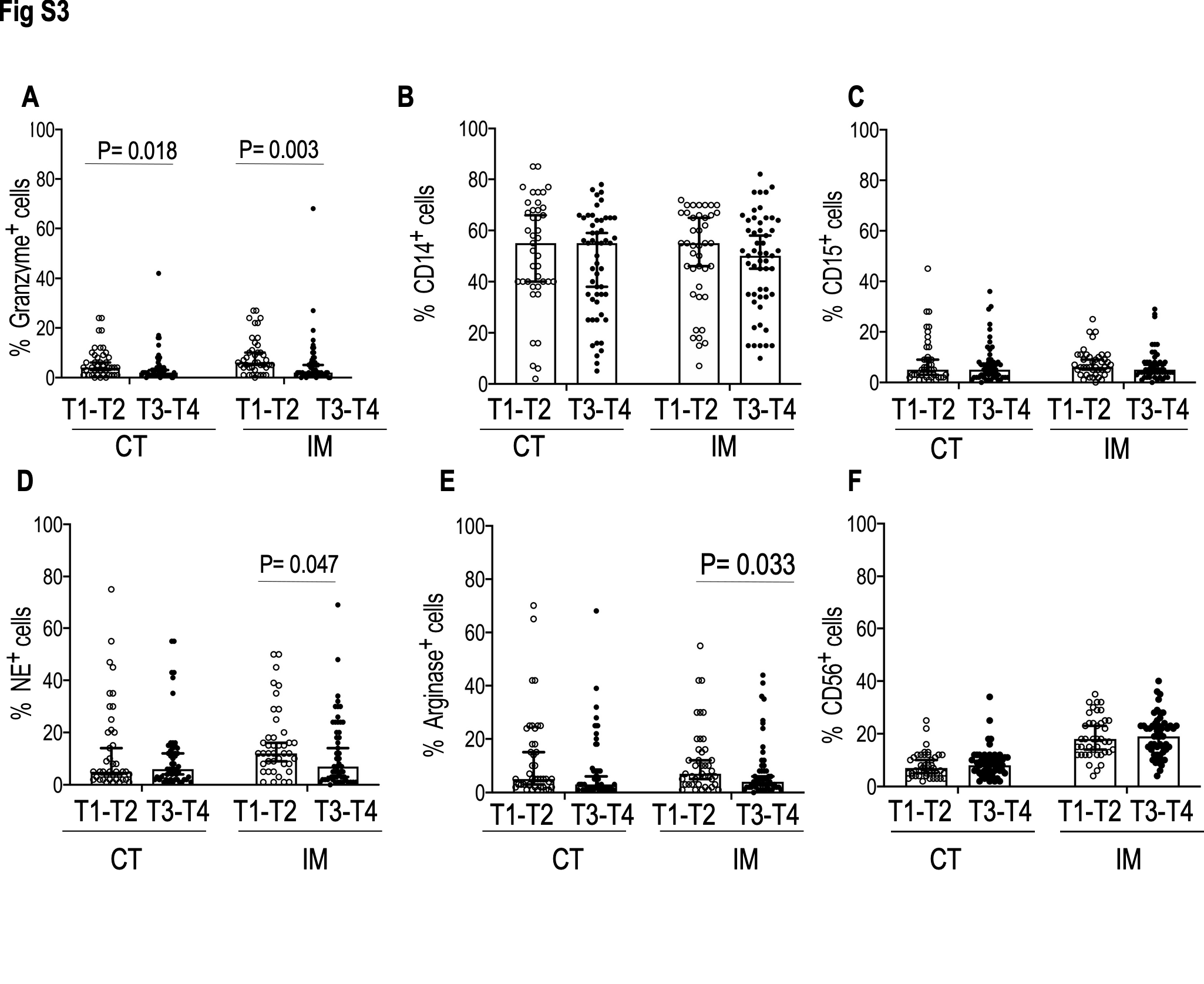

Supplement: S3 Fig — PerkinElmer inForm software was used to enumerate densities of immune cells. Data represented are median with 95% CI. Open circle denotes T1& T2 and the closed circle denotes T3&T4. Two-tailed Mann Whitney test was performed to test statistical significance. T1& T2 (n = 41) and T3&T4 (n = 53). (TIF) [file pone.0242058.s003.tif]

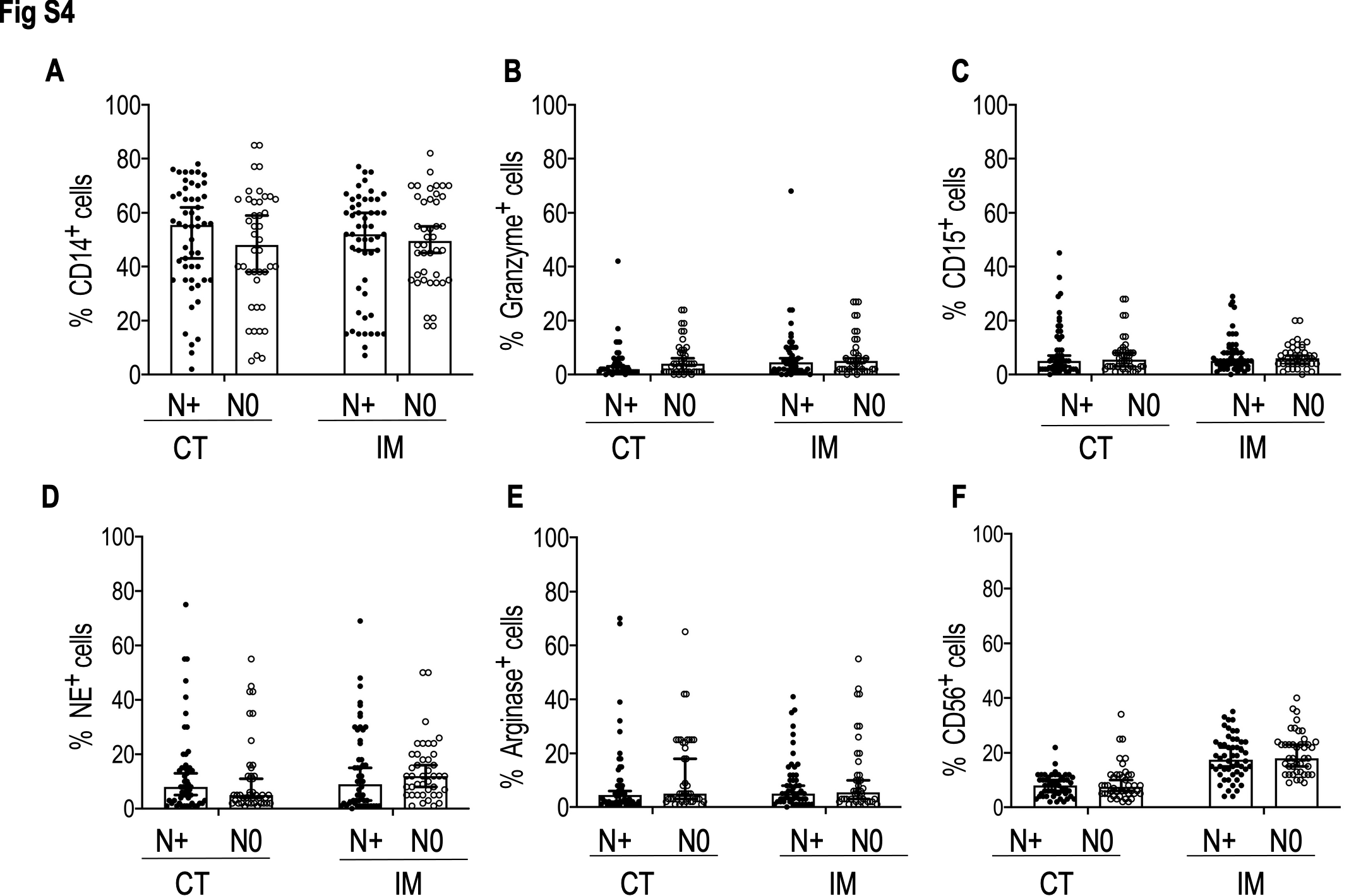

Supplement: S4 Fig — PerkinElmer inForm software was used to enumerate densities of immune cells. Data represented are median with 95% CI. Closed circle denotes N+ and the open circle denotes N0. Two-tailed Mann Whitney test was performed to test statistical significance. N+ (n = 52) and N0(n = 42). (TIF) [file pone.0242058.s004.tif]

**S1Table**

**
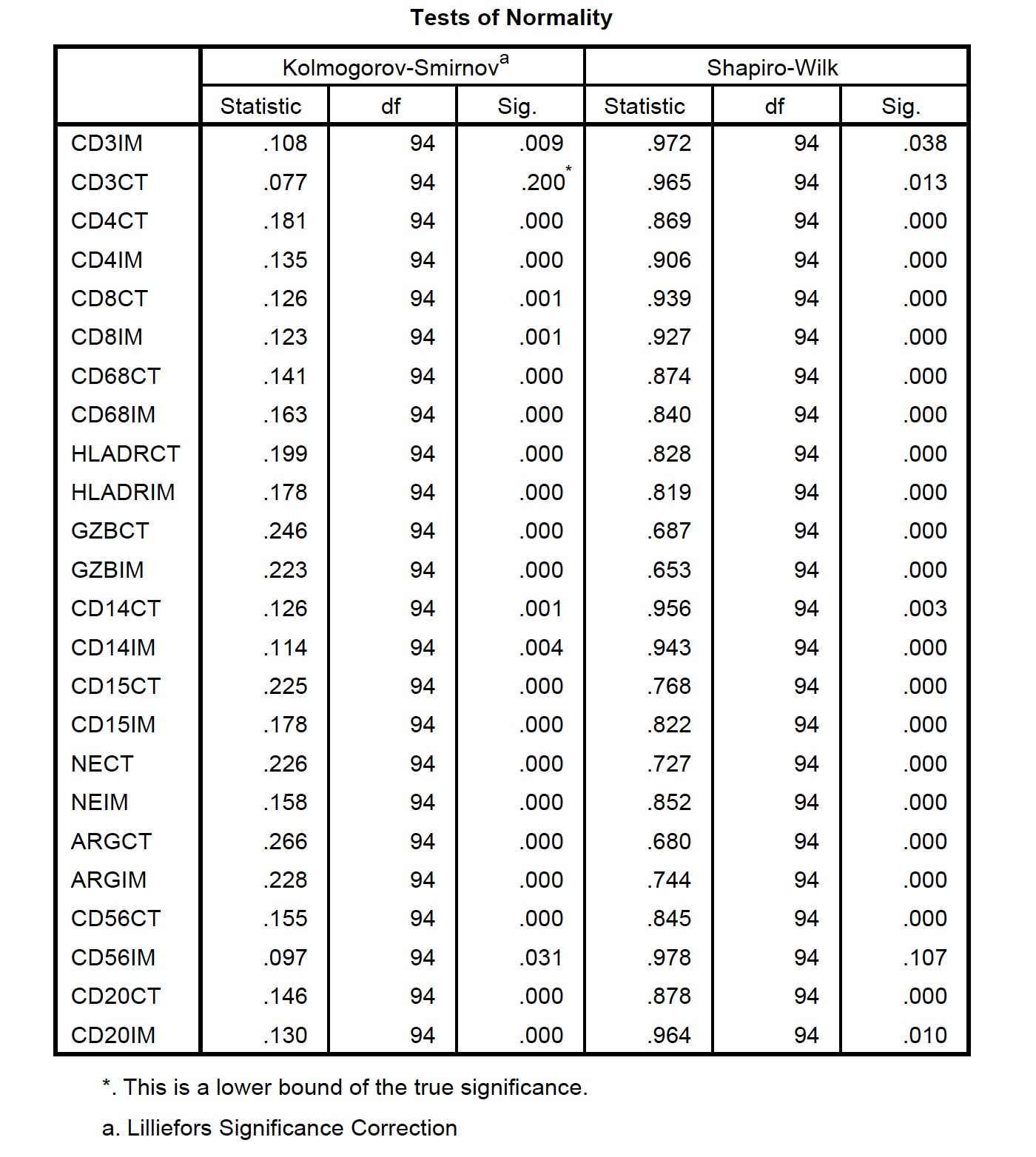
**

Supplement: S1 Table — (DOCX) [file pone.0242058.s005.docx]
