# Supplementary material for: Density of CD3+ and CD8+ cells in gingivo-buccal oral squamous cell carcinoma is associated with lymph node metastases and survival
Source: PLoS One. 2020 Nov 19;15(11):e0242058. doi: 10.1371/journal.pone.0242058 (PMC7676650; doi:10.1371/journal.pone.0242058)
Supplement: Attachment — Submitted filename: Response to Reviewers_Mukherjee etal.doc [file pone.0242058.s007.doc]

**Reviewer 1**

Thank you very much for evaluating our manuscript. We appreciate your insightful comments. We have revised the manuscript accordingly to address the critiques.

**Critique1. The title is intricate and should be rephrased (Density of CD3+ and CD8+ cells in gingiva-buccal oral squamous cell carcinoma is associated with lymph node metastases and survival).**

Response: We have changed the title to “Density of CD3^+^ and CD8^+^ cells in Gingivo-Buccal Oral Squamous Cell Carcinoma is associated with lymph node metastases and survival” as suggested.

**Critique2. Tumor center is more logically be abbreviated TC**

Response: The center of tumor is abbreviated as CT conventionally. Therefore, we have changed “tumor center” in the text to “center of tumor” that is abbreviated as CT.

**Critique3. Abstract need linguistic revision. Also, the statement. “Furthermore, high densities of CD3+ and CD8+ cells in CT resulted in a significantly longer patient survival” should be revised as the term “resulted in” suggests a causal relationship that cannot be determined based on this type of study**.

Response: We have made revisions to the abstract and have removed the phrase -results in.

**Critique 4. Introduction: This was first described by Galon et al. in colorectal cancer [17, 19], where the density of T cells was measured as a “score” both at the invasive tumor margin and tumor center. Subsequently, the consensus immunoscore categorizing inflamed and non-inflamed tumors was validated globally with high clinical relevance [25]. Reference 25 only includes colorectal cancer, thus this statement should be stated as globally can be misinterpreted as to be valid for all types of cancer.**

Response: We have removed the word globally.

**Critique 5. Materials and Methods:
a. Patient tumor tissue: had any of the included patients had prior treatment at TMC? What version of the TNM classification was used? Is the N-status clinically or pathologically determined? Please include references to guidelines**.

Response: None of the patients had prior treatment at TMC. The TMN classification was used according to American Joint Committee on Cancer (AJCC) Cancer staging manual / Union for International Cancer Control (UICC), 7^th^ edition, 2010. The reference has been added.

The N-status was determined pathologically.

We have added the reference to the guidelines.

b**. Slide image analyses: “For each slide, 5 different regions of tumor, including the tumor center and margins, were captured.” Which regions, except tumor center and margins were captured? How many images were assessed for each region? “Immune cell levels were quantified as the percentage of cells with a minimal intensity that was considered positive [35].” It is difficult to grasp this description of how the staining was quantified, please add a more thorough description. How was the reproducibility of scoring between the two observers? It would be informative to include the definitions of immune hot and immune altered phenotypes**.

Response: Five different regions from invasive margin and 5 regions from tumor center were selected by the pathologist in order to cover the entire tumor and the average scores were taken for each of the sites. Images were taken at 10x and 20x magnifications. Only images captured at 20x magnification were analyzed using the InForm 2.4 software and checked by two pathologists (Perkin-Elmer).In a small number of cases where scoring seemed to be inaccurate due to mild background staining (5%), the slides were checked manually by two pathologists and the average score of the independent assessment of the pathologists was considered. We have added this part to the Slide image analysis section.

We have added two sentences about immune hot and immune altered phenotypes of tumors in the introduction.

**Critique 6. Results**:
**a. Patient characteristics: These numbers could have been displayed in a table.**

Response: We have included a table (Table I) to display patient characteristics.

**Critique 7. Discussion: a. “One study has shown that the risk of malignant transformation from leukoplakia is high in oral cancers when the total CD3+cells in the TME are less in contrast to the presence of high density of CD3+ in leukoplakia that did not transform to squamous cell carcinoma [39]” Sentence need linguistic revision.**

Response: We have revised the sentence. One study has shown that patients with leukoplakia without malignant transformation had higher numbers of CD3^+^ cells than patients whose leukoplakia transformed to oral squamous cell carcinoma.

**b. “Our results are in concordance with existing reports [40-42] that pre-existing tumor infiltrating CD8+T cells are strongly associated with improved patient survival.” What is meant by pre-exiciting?**

Response: We have revised the sentence. The presence of a baseline anti-tumor immune response or pre-existing anti-tumor immunity that is associated with high levels of tumor infiltrating CD8^+^T cells is strongly associated with improved patient survival.

**c. “Another study used tissue microarrays (TMA) [43]” There is no information about what was found in this study**.

Response: Another study used tissue microarrays (TMA) to investigate the role of tumor infiltrating cells to predict patient outcome. They found that CD68^+^ macrophages were higher in patients with positive lymph nodes.

**d. “There are no studies with specific or homogeneous sub sites.” This is not true, at least this paper assesses immune infiltrate in OSCC, on whole tissue sections: DOI:10.1038/s41379-018-0019-5.**

Response: We have made changes in the text and have included this reference.

**Critique 8. General: Multivariate analyses would strengthen the study.**

Response: We have performed multivariate analyses. The data is shown in Table 2 and discussed in the Results and Discussion sections.

**Critique 9. For readers not familiar with immune cell markers it would be informative if the type of immune cells recognized by the various markers were specified**

Response: We have provided information about each immune cell marker in the single marker immune-histochemistry section of Materials and Methods.

**Reviewer 2**

Thank you very much for evaluating our manuscript. We appreciate your insightful comments. We have revised the manuscript accordingly to address the critiques.

**Major issues:**

**Critique 1- It would be essential to know the HPV status of the tumors, as HPV infection is known to result in immunologically hot tumors, better response to therapy and improved survival. Hence, if the HPV status of the tumors is unknown, performing e.g. an additional P16 staining would be very important, to rule out that that is a potential confounder in this study**.

Response: We have performed p16 staining to the HPV status. The p16 staining result has been added in the Patient Characteristics section of the Results. S1Fig. shows the p16 staining.

**Critique 2- Why was the relationship of these 12 markers with M stage not investigated, but only with T and N stage?**

Response: All 94 cases were M0 at diagnosis. We have mentioned this in Table I (Patient Characteristics).

**Critique 3- Regarding the statistical analyses: was the data normally distributed, i.e. was a test for normality performed? If not, that would be necessary. And if the data is not normally distributed, medians instead of means should be presented in the text and figures, and e.g. Mann-Whitney tests should be performed instead of T-tests.**

Response: We have revised the statistical analyses section in Materials and Methods. Distribution pattern for expression of all immune cell markers was checked by Shapiro-Wilk normality test [p<0.05 (CI 95)]. (Data provided in Table S1 ). Mann-Whitney test was performed to compare the expression level of each immune cell marker between node-positive vs negative groups or tumor size T1-T2 vs T3-T4 groups. Expression of all immune cell markers except CD56 expression in invasive margin did not follow normal distribution. The graphs in Figures 2 and 3 show median values with 95% CI.

**Critique 4- How do the authors biologically explain the differences in findings between IM and CT? (e.g. improved survival was only observed in patients with high densities of CD3+ and CD8+ cells in the CT, but no such association was seen in the IM)**

Response: This may be likely due to productive anti-tumor immune response as a result of the ability of cytotoxic T cells to infiltrate CT and establish physical contact with tumor cells for tumor killing. On the other hand, cytotoxic T cells at the invasive margin may have the ability to mount anti-tumor response but tumor cells escape killing by hindering T cell infiltration as proposed by Galon and Bruni. We have added these sentences to the discussion.

**Critique 5- Paragraph ‘Association of Tumor Immunoscore with Lymph Node Metastasis’: why was this association of Tumor Immunoscore not also investigated with T and M stage as well?**

Response: Immunoscore quantifies both CD3^+^ and CD8^+^ cells at the invasion margin and center of tumors. We have replaced the word immunoscore with immune cell marker expression score as we quantified CD3^+^ and CD8^+^ markers at the invasive margin and center of tumor separately. We have performed immune cell marker expression score for center of tumor for T stage (Figure 4B). All 94 cases were M0. Subsequently, 5 patients developed distant metastasis within one year of diagnosis.

**Critique 6 - Results section, sentence ‘The association of markers’ expression (CT) with node status was further evaluated through ‘line of best fit’ method.’ Why only CT, and not IM as well?**

Response: We have performed Line of Best Fit analyses for IM (Figure 4A).

**Minor issues:**

**Introduction:**

**Critique7- ‘The morbidity and mortality associated with OSCC-GB remains poor’ quantify poor**

Response: We have revised the sentence. Despite advances made in treatment modalities, locoregional recurrence is the primary cause of treatment failure in advanced stages of the disease with a dismal 5-year survival rate of 5-15%.

**Critique 8- ‘The consensus immunoscore’ only in colorectal cancer it has recently reached consensus, in most other cancer types not yet, this needs rephrasing.**

Response: We have rephrased the sentence to include colorectal cancer.

**Critique 9- ‘Grading and staging were performed according to the WHO classification of tumors and UICC TNM classification’ missing the references here.**

Response: The TMN classification was used according to American Joint Committee on Cancer (AJCC) Cancer staging manual / Union for International Cancer Control (UICC), 7^th^ edition, 2010. The reference has been added

**Critique 10 - ‘All patients received standard-of-care treatment according to National Comprehensive Cancer Network (NCCN) guidelines’ missing references.**

Response: We have added references to the guidelines.

**Methods:**
**Critique 11- ‘After washing, peroxidase blocking was carried out’ with what were the washing steps performed?**

Response: Washing was done with wash buffer.

**Critique 12-The rationale behind the selection of these 12 markers is lacking, please add this.**

Response: We selected immune markers related to adaptive and innate immunity. We have added this sentence in the single marker immunohistochemistry section.

**Critique 13 Were consecutive slides used for the 12marker single IHC stainings?**

Response: Consecutive slides were used for the 12marker single IHC staining. We added this sentence in the single marker immunohistochemistry section.

**Critique 14 -at 10x and 20x magnification’ it is not clear what the authors mean by this. Were images of all tumors taken at both 10x and 20x magnification? Which images were used for the analyses?**

Response: The images were taken at 10x and 20x magnification. However, images at 20x were used for analysis**.** We have mentioned this in the Image Analysis section of Materials and Methods.

**Critique 15- ‘For each slide, 5 different regions of tumor, including the tumor center and margins, were captured’ 5 regions of both CT and IM together (and if so, how many of which), or 5 regions of CT and 5 regions of IM? And how were the regions selected? This needs to be elaborated.**

Response: Five different regions from invasive margin and 5 regions from tumor center were selected by the pathologist in order to cover the entire tumor and the average scores were taken for each of the sites. Images were taken at 10x and 20x magnifications. Only images captured at 20x magnification were analyzed using the InForm 2.4 software (Perkin-Elmer).

**Critique 16- ‘The final score was an average from independent assessments of histopathological images by of two onco-pathologists.’ Was the score obtained using InForm software used, or the scoring performed by the pathologists? It is unclear which data was obtained using InForm, and which data from scoring by the pathologists, also in the results section.**

Response: Images were analyzed using the InForm 2.4 software and checked by two pathologists (Perkin-Elmer). In a small number of cases where scoring seemed to be inaccurate due to mild background staining (5%), the slides were checked manually by two pathologists and the average score of the independent assessment of the pathologists was considered. We have added this part to the Slide image analysis section.

**Critique 17- ‘Each patient was re-contacted and relevant data collected for at least two years by telephone calls or during their follow up visits to the hospital.’ What was the relevant data that was collected?**

Response: Each patient was re-contacted and relevant data such as complaints, general conditions, symptoms and history of recurrence were collected. If there was any complaint that patients had, they were advised to immediately visit the hospital, or get medical advice from a hospital near their home.

**Statistical analysis:**

**Critique 18 ‘explanatory variable’ such as?**

We have revised the statistical analyses section of Materials and Methods and removed ‘explanatory variable’ such as.

**Critique 19 - ‘Multiple testing correction was done, wherever appropriate’ how was this correction performed? And when was this considered ‘appropriate’? Please elaborate.**

We have revised the statistical analyses section of Materials and Methods and removed ‘Multiple testing correction was done, wherever appropriate’

**Critique 20 - ‘K-Nearest Neighbors (KNN) based supervised machine learning algorithm was used to predict any plausible classification between the expression of immune cell markers and node positivity.’ Why not also between the expression of immune cell markers and T stage?**

We have performed K-Nearest Neighbors (KNN) based supervised machine learning algorithm to predict any plausible classification between the expression of immune cell markers and tumor size (T stage). The table in Figure 4C shows the analysis.

**Results:**

**Critique 21 - Be consistent in fully writing out the numbers or not. Here, not writing them full out would be clearer (e.g. ‘Ninety-five percent’ as 95%).**

Response**:** We have removed ninety-five percent.

**Critique 22 - The 95% confidence intervals are missing behind each p-value, please add these.**

Response: We have added 95% CI behind P values.

**Critique 23 - ‘No statistically significant difference (p>0.05) in the expression of Granzyme B, CD68, neutrophil elastase, CD15, CD14, Arginase1, CD56 were found between the T1&T2 and T3&T4 groups in both the CT and IM (S2Fig).’ add the underlined words. Remove ‘CD68’ in this sentence, as in supplemental figure 2 and in the following sentence it becomes apparent that there are differences in CD68 expression between the two groups**.

Response: We have removed CD68 from the sentence. After performing Mann Whitney as recommended, there were significant differences in expression Granzyme B+ cells between T1-T2 vs T3-T4 in both center of tumors (P= 0.018 (95% CI) and invasive margins (P=0.003 95% CI). There were significant differences in expression of Neutrophil Elastase (P=0.047 95% CI) and Arginase P=0.033 95% CI) in invasive margins between T1-T2 vs T3-T4 groups. We have added these sentences in the Results section- Association of immune cell infiltrates at the invasive margin and center of tumors with tumor size and lymph node metastasis.

**Discussion:**

**Critique 24 - ‘HLA-DR is recognized as a marker of T cell activation [48]’ that is not correct, HLA-DR is required for T cell activation, but is not in itself a marker of T cell activation.**

Response: We have rephrased the sentence to HLA-DR is expressed on activated T cells.

**Critique 25 - ‘All patients were uniformly treated’ how is that? Please specify.**

Response: Following surgical resection of tumors, patients received adjuvant radiotherapy/chemotherapy based on pathological parameters (pTNM staging) in accordance with NCCN guidelines. We have added this to the discussion section.

**Critique 26 - ‘The results in different studies are variable because of the non-specific nature of staining. Therefore, CD68 cannot be considered a marker of significance.’ The statement that CD68 cannot be considered a marker of significance is not supported by many recent studies, that have found that high CD68 expression is frequently associated with better survival and response to (immune)therapies.**

Response: We have removed the two sentences- The results in different studies are variable because of the non-specific nature of staining. Therefore, CD68 cannot be considered a marker of significance - CD68 cannot be considered a marker of significance.

We have replaced these sentences with the following.CD68 is a pan macrophage marker that does not discriminate between tumoricidal M1 macrophages and anti-inflammatory expressing M2 macrophages. In Oral Squamous Cell Carcinoma expression of CD163 significantly correlated with overall survival while expression of CD68 did not. A meta-analysis study in Oral Squamous Cell Carcinoma showed that CD163 is a more reliable prognostic marker than CD68. The CD68^+^ cells in our study may be pro-inflammatory M1 macrophages. Therefore, staining of a co-stimulatory molecule CD80 expressed by pro-inflammatory macrophages along with scavenger receptor CD163 is necessary to characterize these macrophages. Interestingly, Pinto et al showed that CD80^+^ cells were more abundant in intra-tumoral region and invasive front of less invasive T1 stage of colorectal cancer.

**Critique 27 - ‘Although we found high densities CD4+ cells in the tumors associated with lymph negative disease, this marker is expressed on T cells as well as macrophages and monocytes [47]. Therefore, staining for CD4 expression alone does not distinguish between these cells and CD4+ T cells.’ What is the relevance of these sentences related to this study? They currently seem abundant, and would better be removed.**

Response: We have removed these sentences.

**Figures:
Critique 28 - Figure 4A: unclear what the axes mean, what are the units of the X-axes and Y-axes? This figure needs a more elaborate figure legend.**

Response: We have added X-axes and Y-axes labels in Figure 4A.

**Critique 29 Figure 5: write in each Kaplan-Meier graphs behind ‘High’ and ‘Low’ ‘n=……’ for each graph line respectively**

Response: We have added n values behind High and Low values in each Kaplan Meir graphs in Figure 5.

**Critique 30 Other comments:
There are quite some double spaces and interpunction errors (e.g. dot instead of comma in an enumeration). Please thoroughly go through the manuscript to remove these errors.
Some additional formulation mistakes:**
**Page 3: ‘The composition of the tumor immune microenvironment’**

**Page 4: ‘such as chromosomal instability and mutations’ replace mutations by mutation burden**

**Page 4: ‘Immuno-histochemical’ remove dash, consistently without dash**

**Page 4: ‘clinical and pathology details of each patient were collected from hospital records’**

**Page 6: ‘assessments of histopathological images by of two onco-pathologists.’ Remove of**

**Page 8: ‘Patients with high percentages of CD3+, CD8+ and CD68 + cells at the tumor centers and invasive margins were more frequently lymph node metastasis negative.’**

**Page 8: ‘while a high HLA-DR expression at the invasive edge was associated with lymph node metastasis.’ Remove ‘a’**

**Page 9: ‘Fig 4B showed that ‘High’ and ‘Intermediate’ immunoscore are significantly (p<0.05) increased in patients with node negative status compare with node positive patients.’**

**Page 10: ‘However, there are no studies where CD3+ cell count has been correlated with tumor size.’**

**Page 11: ‘The lack of T cell infiltration in the tumor core could indicate immunological ignorance - inability of adaptive immunity to recognize tumors [46].’**

**Page 11: ‘In our study high CD68 density had association with lymph node negative disease.**’

Response: We have corrected the errors.

**Critique 31 Figures 1, 2, 3: make these figures in portrait, not landscape, orientation**

Response: We have changed the orientation of Figures 1, 2 and 3 to portrait orientation.

**Critique 32 Supplemental figure 1: write the name of the marker in the image, instead of the numbers, that makes it clearer to the reader.**

Response: We have written the names of the markers in the image.
