# Supplementary material for: Density of CD3+ and CD8+ cells in gingivo-buccal oral squamous cell carcinoma is associated with lymph node metastases and survival
Source: PLoS One. 2020 Nov 19;15(11):e0242058. doi: 10.1371/journal.pone.0242058 (PMC7676650; doi:10.1371/journal.pone.0242058)
Supplement: Attachment — Submitted filename: Reviewer comments to PONE-D-20-17762.docx [file pone.0242058.s006.docx]

**Reviewer comments to ‘Association of densities of CD3+ and CD8+ cells in tumors with lymph node metastasis and survival in Gingivo-buccal Oral Squamous Cell Carcinoma’**

**Overall impression:**

Mukherjee et al investigated the relationship between T and N stage and the expression of 12 markers (CD3, CD8, CD4, Granzyme B, CD68, neutrophil elastase, HLADR, CD15, CD14, arginase1, CD56 and CD20) in the invasive margin (IM) and tumor center (CT) in a cohort of 94 gingiva-buccal OSCC patients. They found that higher densities of CD3+ cells were associated with lower T stage and, and higher densities of both CD3+ and CD8+ cells were associated with the absence of lymph node metastases and better survival.

They performed an elaborate study of 12 immune related markers, in a gingiva-buccal OSCC dedicated cohort. The research question is straightforward, the findings are of interest, however there are some points that need to be addressed.

**Major issues:**

- It would be essential to know the HPV status of the tumors, as HPV infection is known to result in immunologically hot tumors, better response to therapy and improved survival. Hence, if the HPV status of the tumors is unknown, performing e.g. an additional P16 staining would be very important, to rule out that that is a potential confounder in this study.
- Why was the relationship of these 12 markers with M stage not investigated, but only with T and N stage?
- Regarding the statistical analyses: was the data normally distributed, i.e. was a test for normality performed? If not, that would be necessary. And if the data is not normally distributed, medians instead of means should be presented in the text and figures, and e.g. Mann-Whitney tests should be performed instead of T-tests.
- How do the authors biologically explain the differences in findings between IM and CT? (e.g. improved survival was only observed in patients with high densities of CD3+ and CD8+ cells in the CT, but no such association was seen in the IM)
- Paragraph ‘Association of Tumor Immunoscore with Lymph Node Metastasis’: why was this association of Tumor Immunoscore not also investigated with T and M stage as well?
- Results section, sentence ‘The association of markers’ expression (CT) with node status was further evaluated through ‘line of best fit’ method.’ Why only CT, and not IM as well?

**Minor issues:**

Introduction:

- ‘The morbidity and mortality associated with OSCC-GB remains poor’ quantify poor
- ‘The consensus immunoscore’ only in colorectal cancer it has recently reached consensus, in most other cancer types not yet, this needs rephrasing.
- ‘Grading and staging were performed according to the WHO classification of tumors and UICC TNM classification’ missing the references here
- ‘All patients received standard-of-care treatment according to National Comprehensive Cancer Network (NCCN) guidelines’ missing references

Methods:

- ‘After washing, peroxidase blocking was carried out’ with what were the washing steps performed?
- The rationale behind the selection of these 12 markers is lacking, please add this.
- Were consecutive slides used for the 12 marker single IHC stainings?
- ‘at 10x and 20x magnification’ it is not clear what the authors mean by this. Were images of all tumors taken at both 10x and 20x magnification? Which images were used for the analyses?
- ‘For each slide, 5 different regions of tumor, including the tumor center and margins, were captured’ 5 regions of both CT and IM together (and if so, how many of which), or 5 regions of CT and 5 regions of IM? And how were the regions selected? This needs to be elaborated.
- ‘The final score was an average from independent assessments of histopathological images by of two onco-pathologists.’ Was the score obtained using InForm software used, or the scoring performed by the pathologists? It is unclear which data was obtained using InForm, and which data from scoring by the pathologists, also in the results section.
- ‘Each patient was re-contacted and relevant data collected for at least two years by telephone calls or during their follow up visits to the hospital.’ What was the relevant data that was collected?

Statistical analysis:

- ‘explanatory variable’ such as?
- ‘Multiple testing correction was done, wherever appropriate’ how was this correction performed? And when was this considered ‘appropriate’? Please elaborate.
- ‘K-Nearest Neighbors (KNN) based supervised machine learning algorithm was used to predict any plausible classification between the expression of immune cell markers and node positivity.’ Why not also between the expression of immune cell markers and T stage?

Results:

- Be consistent in fully writing out the numbers or not. Here, not writing them full out would be clearer (e.g. ‘Ninety-five percent’ as 95%).
- The 95% confidence intervals are missing behind each p-value, please add these.
- ‘No statistically significant difference (p>0.05) in the expression of Granzyme B, CD68, neutrophil elastase, CD15, CD14, Arginase1, CD56 were found between the T1&T2 and T3&T4 groups in both the CT and IM (S2Fig).’ add the underlined words. Remove ‘CD68’ in this sentence, as in supplemental figure 2 and in the following sentence it becomes apparent that there are differences in CD68 expression between the two groups.

Discussion:

- ‘HLA-DR is recognized as a marker of T cell activation [48]’ that is not correct, HLA-DR is required for T cell activation, but is not in itself a marker of T cell activation.
- ‘All patients were uniformly treated’ how is that? Please specify
- ‘The results in different studies are variable because of the non-specific nature of staining. Therefore, CD68 cannot be considered a marker of significance.’ The statement that CD68 cannot be considered a marker of significance is not supported by many recent studies, that have found that high CD68 expression is frequently associated with better survival and response to (immune)therapies.
- ‘Although we found high densities CD4+ cells in the tumors associated with lymph negative disease, this marker is expressed on T cells as well as macrophages and monocytes [47]. Therefore, staining for CD4 expression alone does not distinguish between these cells and CD4+ T cells.’ What is the relevance of these sentences related to this study? They currently seem abundant, and would better be removed.

Figures:

- Figure 4A: unclear what the axes mean, what are the units of the X-axes and Y-axes? This figure needs a more elaborate figure legend.
- Figure 5: write in each Kaplan-Meier graphs behind ‘High’ and ‘Low’ ‘n=……’ for each graph line respectively

**Other comments:**

There are quite some double spaces and interpunction errors (e.g. dot instead of comma in an enumeration). Please thoroughly go through the manuscript to remove these errors.

Some additional formulation mistakes:

Page 3: ‘The composition of the tumor immune microenvironment’

Page 4: ‘such as chromosomal instability and mutations’ replace mutations by mutation burden

Page 4: ‘Immuno-histochemical’ remove dash, consistently without dash

Page 4: ‘clinical and pathology details of each patient were collected from hospital records’

Page 6: ‘assessments of histopathological images by of two onco-pathologists.’ Remove of

Page 8: ‘Patients with high percentages of CD3+, CD8+ and CD68 + cells at the tumor centers and invasive margins were more frequently lymph node metastasis negative.’

Page 8: ‘while a high HLA-DR expression at the invasive edge was associated with lymph node metastasis.’ Remove ‘a’

Page 9: ‘Fig 4B showed that ‘High’ and ‘Intermediate’ immunoscore are significantly (p<0.05) increased in patients with node negative status compare with node positive patients.’

Page 10: ‘However, there are no studies where CD3+ cell count has been correlated with tumor size.’

Page 11: ‘The lack of T cell infiltration in the tumor core could indicate immunological ignorance - inability of adaptive immunity to recognize tumors [46].’

Page 11: ‘In our study high CD68 density had association with lymph node negative disease.’

Figures 1, 2, 3: make these figures in portrait, not landscape, orientation

Supplemental figure 1: write the name of the marker in the image, instead of the numbers, that makes it clearer to the reader.

Thank you.
