# Supplementary material for: Density of CD3+ and CD8+ cells in gingivo-buccal oral squamous cell carcinoma is associated with lymph node metastases and survival
Source: PLoS One. 2020 Nov 19;15(11):e0242058. doi: 10.1371/journal.pone.0242058 (PMC7676650; doi:10.1371/journal.pone.0242058)
Supplement: Attachment — Submitted filename: Reviewer comments to PONE-D-20-17762.R1.docx [file pone.0242058.s008.docx]

**Reviewer comments to PONE-D-20-177.R1**

The authors have done a nice job incorporating the comments.

It is interesting to see that only 4 out of 94 tumors were (equivocal) HPV positive (using P16 IHC as a surrogate marker for HPV), in order to put this into perspective, it would be relevant if the authors could add the percentage of HPV positive tumors as generally found in literature for OSCC.

One other comment that remains:

Critique 24 - ‘HLA-DR is recognized as a marker of T cell activation [48]’ that is not

correct, HLA-DR is required for T cell activation, but is not in itself a marker of T cell

activation.

Response authors: We have rephrased the sentence to HLA-DR is expressed on activated T cells.

Reviewer: HL-DR is not expressed on T cells, but on the antigen presenting cells. Binding of T cells with their TCR to the respective HL-DR molecule on the APC, can result in T cell activation. This sentence needs rewriting.
